# Gene expression and alternative splicing dynamics are perturbed in female head transcriptomes following heterospecific copulation

**DOI:** 10.1186/s12864-021-07669-0

**Published:** 2021-05-18

**Authors:** Fernando Diaz, Carson W. Allan, Therese Ann Markow, Jeremy M. Bono, Luciano M. Matzkin

**Affiliations:** 1 Department of Entomology, University of Arizona, Tucson, AZ USA; 2 Cinvestav UGA-Langebio, Irapuato, Guanajuato, Mexico; 3 Division of Biological Sciences, Section of Cell and Developmental Biology, University of California, San Diego, California, USA; 4 Department of Biology, University of Colorado Colorado Springs, Colorado Springs, USA; 5 BIO5 Institute, University of Arizona, Tucson, AZ USA; 6 Department of Ecology and Evolutionary Biology, University of Arizona, Tucson, AZ USA

**Keywords:** Speciation, Postmating response, Alternative splicing, Intron retention, RNA-seq, Head transcriptomes, *D. mojavensis*, *D. arizonae*

## Abstract

**Background:**

Despite the growing interest in the female side of copulatory interactions, the roles played by differential expression and alternative splicing mechanisms of *pre-RNA* on tissues outside of the reproductive tract have remained largely unknown. Here we addressed these questions in the context of con- vs heterospecific matings between *Drosophila mojavensis* and its sister species, *D. arizonae*. We analyzed transcriptional responses in female heads using an integrated investigation of genome-wide patterns of gene expression, including differential expression (*DE*), alternative splicing (*AS*) and intron retention (*IR*).

**Results:**

Our results indicated that early transcriptional responses were largely congruent between con- and heterospecific matings but are substantially perturbed over time. Conspecific matings induced functional pathways related to amino acid balance previously associated with the brain’s physiology and female postmating behavior. Heterospecific matings often failed to activate regulation of some of these genes and induced expression of additional genes when compared with those of conspecifically-mated females. These mechanisms showed functional specializations with *DE* genes mostly linked to pathways of proteolysis and nutrient homeostasis, while *AS* genes were more related to photoreception and muscle assembly pathways. *IR* seems to play a more general role in *DE* regulation during the female postmating response.

**Conclusions:**

We provide evidence showing that *AS* genes substantially perturbed by heterospecific matings in female heads evolve at slower evolutionary rates than the genome background. However, *DE* genes evolve at evolutionary rates similar, or even higher, than those of male reproductive genes, which highlights their potential role in sexual selection and the evolution of reproductive barriers.

**Supplementary Information:**

The online version contains supplementary material available at 10.1186/s12864-021-07669-0.

## Background

Sexual reproduction involves a set of coupled interactions affecting the performance of both sexes, such as those involved in mate-recognition, male courtship and female postcopulatory responses [1]. Females undergo a complex process of physiological changes after mating called the postmating response, induced by biochemical interactions between ejaculate components transferred during copulation and female molecules in the reproductive tract [2, 3]. The role of seminal fluid proteins in the female postmating response has been well characterized in *Drosophila* species, and hundreds of male-derived proteins have now been identified in other taxa [4–7]. The female side has remained more elusive and very little is known about the downstream effector genes that mediate the female postmating response, particularly those occurring outside of the female reproductive tract (e.g. genes related to female behavior).

Although transcriptional changes induced by con- or heterospecific matings have been explored in a number of species [8–16], most of these studies have not considered alternative splicing (*AS*) as an additional mechanism by which genes responsible for postmating changes might be regulated [17]. Differential regulation of spliced isoforms created by different combinations of exons (or intron retention, *IR*) from the same genomic loci may have substantial functional consequences not reflected in gene expression [18], which can uncover additional mechanisms within the complexity of molecular reproductive interactions. Recent comparisons of *Drosophila* species show that *AS* diversification contributes to lineage-specific adaptation [19], with sex-biased splicing and *IR* rates [20] in several tissues including the brain [18], suggesting that this mechanism might be important in female behavioral responses.

Changes induced by heterospecific matings can compromise gametic interactions during the fertilization process leading to postmating prezygotic or *PMPZ* isolation [2, 21]. Moreover, conspecific matings are often accompanied by a set of behavioral changes, such as those involved in female receptivity, exploration, diet and oviposition [22–27]. If altered by mating with a heterospecific male, these behavioral changes can compromise the mating outcome, leading to reproductive barriers. The transcriptional bases of these responses are more likely located in tissues of the central nervous system [28]. In fact, in *D. melanogaster* the seminal fluid protein (SFP) Sex Peptide (SP), is not only one of the main triggers of the female postmating response, but is also gradually released into the hemolymph by cleavage [29–31], suggesting important and lasting changes outside of the reproductive tissues. Sex Peptide interacts with a sex peptide receptor (SPR) in the female, which is expressed in both reproductive organs and the nervous system [32]. Consistent with this, transcriptional changes associated with behavioral and photoreception pathways have been detected in female heads after conspecific mating [25, 33].

It is well known that interacting male and female reproductive genes evolve rapidly [34], however it is unclear whether genes governed by *AS* dynamics or expressed outside of the reproductive tissues follow this evolutionary path. We addressed these questions by exploring head transcriptomes in con- vs heterospecific matings between *Drosophila mojavensis* and *D. arizonae*. Perturbation of the female transcriptional response by heterospecific matings was first demonstrated in female reproductive tracts of *Drosophila mojavensis* when mated to *D. arizonae* males [8]. These species diverged ~ 0.5Mya [35] and display strong *PMPZ* isolation as fertilization success is reduced after heterospecific matings [21, 36]. Although heterospecific matings occur in both directions [37], transcriptional responses have not been previously explored in *D. arizonae* females. Here we implemented an integrated approach to explore the conspecific context of each species and demonstrate that the postcopulatory response involves functionally different roles played by *DE*, *AS* and *IR* dynamics. These responses are substantially perturbed by heterospecific matings and some of these genes evolve rapidly.

## Results

### Pervasive perturbation of DE and AS following heterospecific matings

**Fig. 1:**
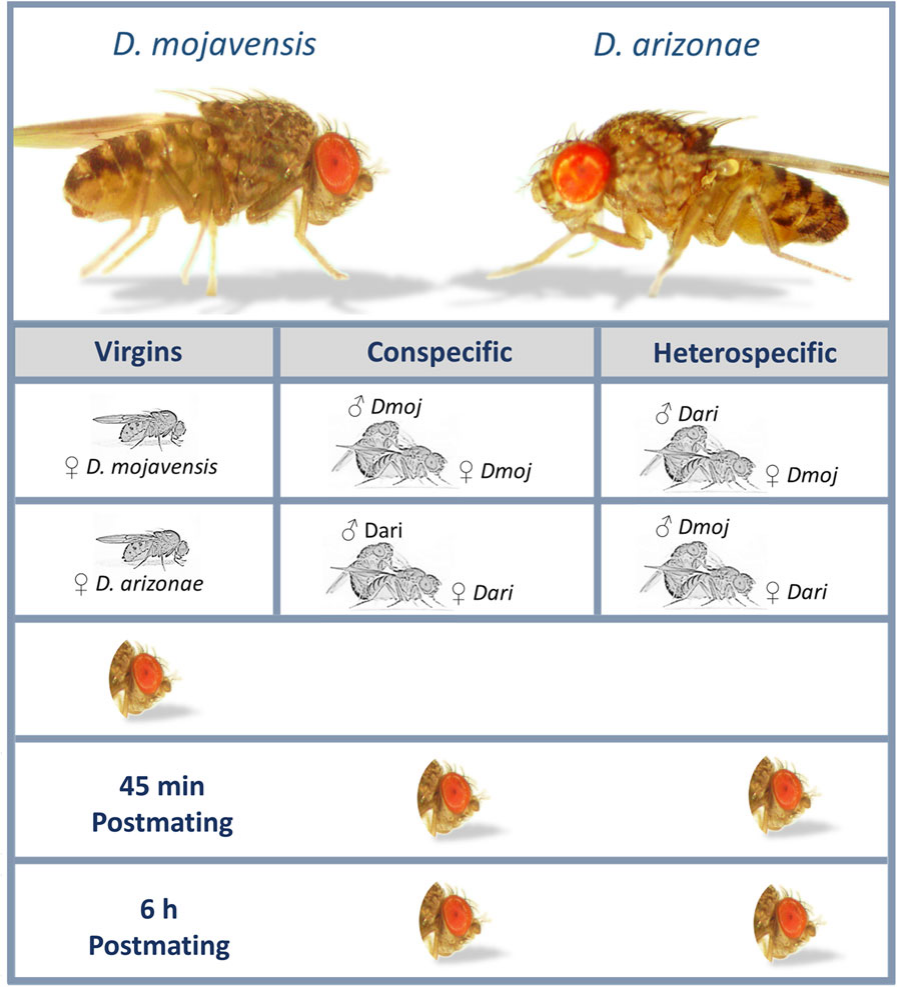
Experimental design for con- and heterospecific matings between *D. mojavensis* and *D. arizonae*. RNA-seq libraries were constructed for head tissues of virgins, con- and heterospecifically-mated females at 45 min and 6 h postmating using three biological replicates

**Fig. 2:**
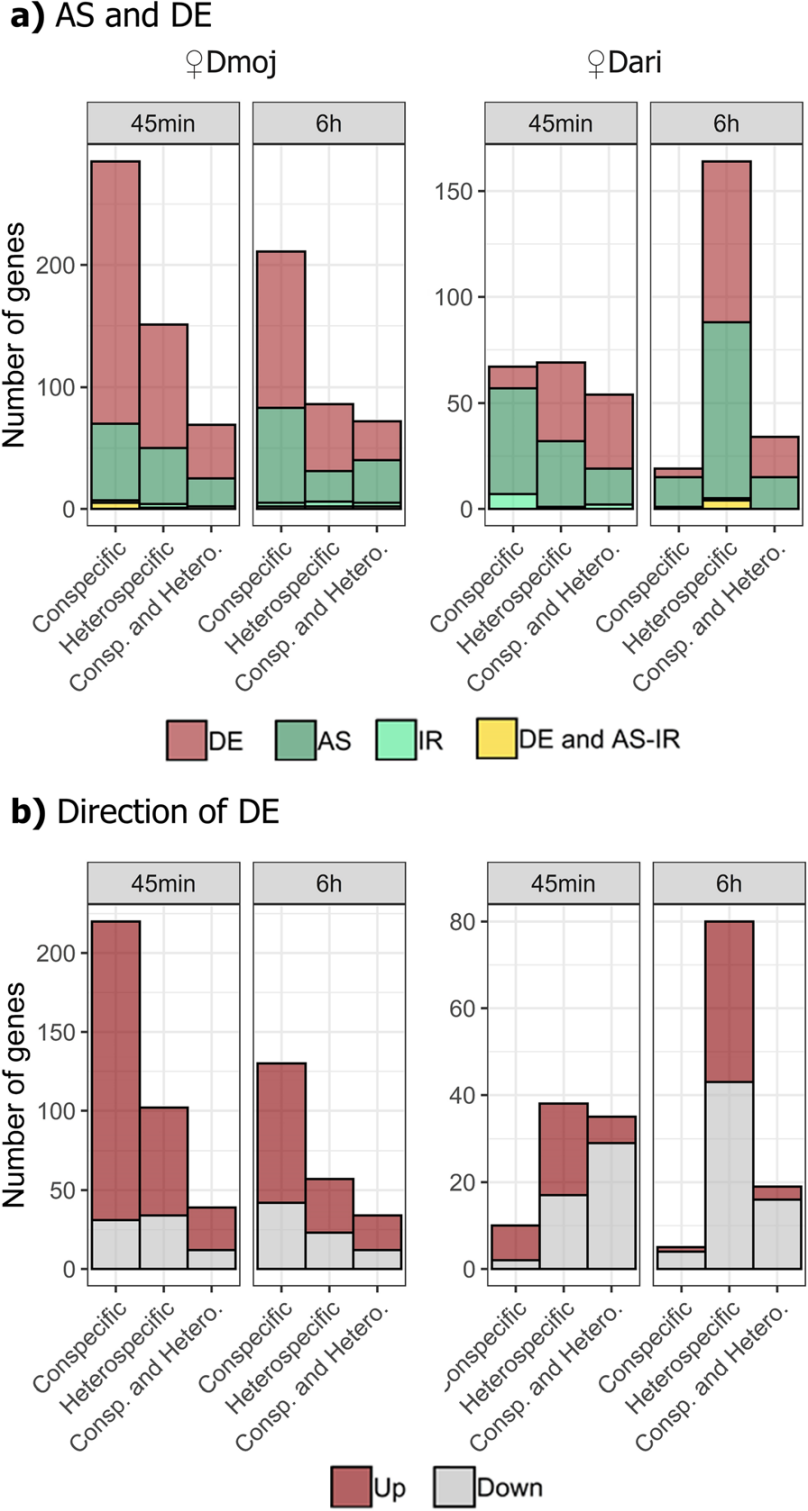
Female transcriptional responses following con- and heterospecific matings between *D. mojavensis* and *D. arizonae*. All comparisons were performed between head transcriptomes of mated and virgin females using three biological replicates. **a** Number of significant *DE* and *AS* genes (including *IR*). **b** Number of significantly down- vs up-regulated *DE* genes. The bars indicate the number of significant genes (*FDR* corrections following global α of: *DE* = 0.05, *AS* = 0.01 and *IR* = 0.05) exclusive to con- or heterospecific matings, as well as their overlap, for crosses involving *D. mojavensis* females (*♀Dmoj*) and *D. arizonae* females (*♀Dari*) at 45 min and 6 h postmating

**Fig. 3:**
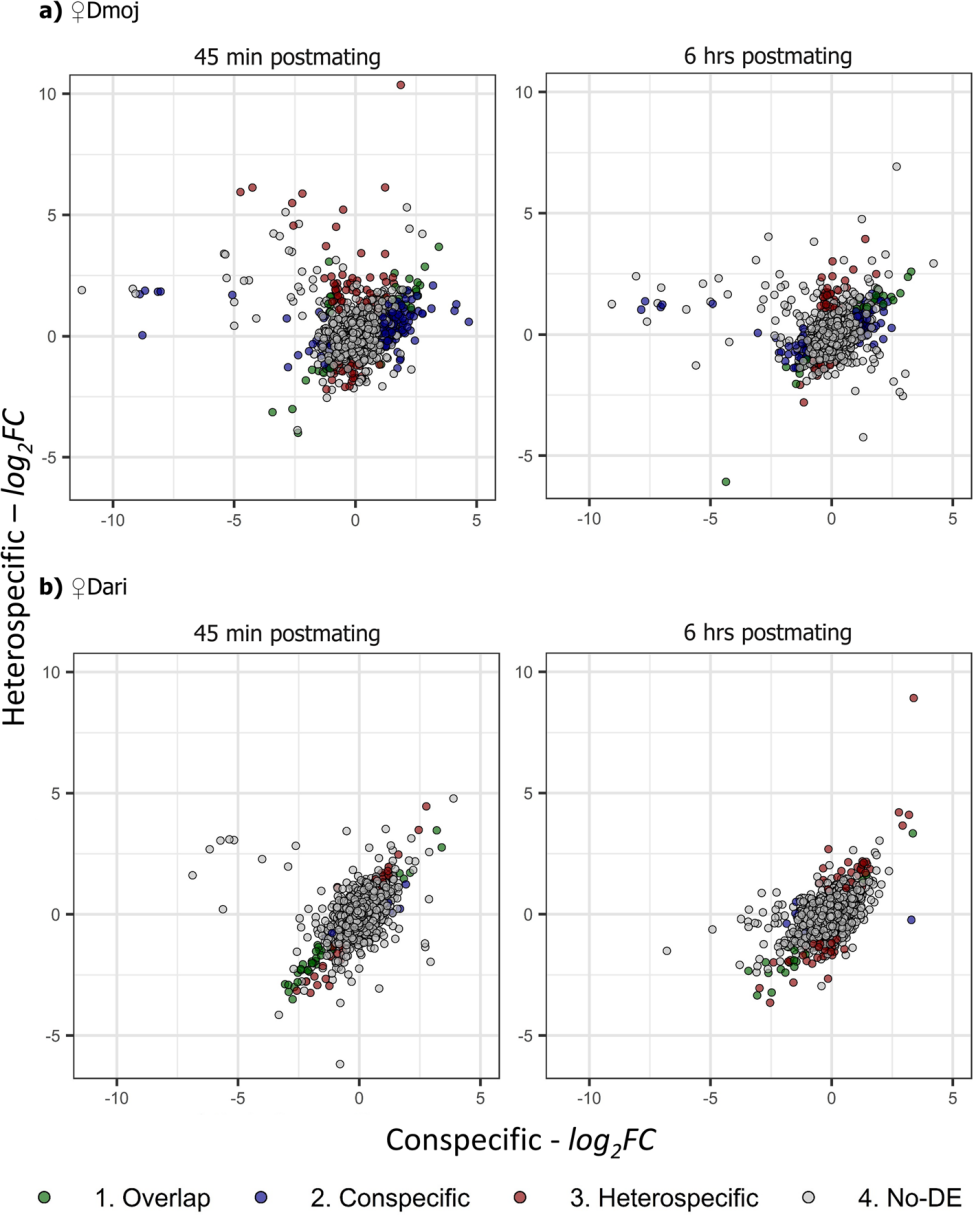
Expression fold changes in head transcriptomes of con- and heterospecifically-mated females of *D. mojavensis* and *D. arizonae*. All *DE* comparisons were between mated and virgin females using three biological replicates. The scatterplots show fold changes (*log*_*2*_*FC*) in relative gene expression and statistical significance of *DE* genes for crosses involving **a** *D. mojavensis* females (*♀Dmoj*) and **b** *D. arizonae* females (*♀Dmoj*) at 45 min and 6 h postmating. Genes differentially expressed in both con- and heterospecific matings are indicated in green, while blue points indicate those exclusive to conspecific matings and red points indicate those exclusive to heterospecific matings (*FDR* corrections following global α of: *DE* = 0.05)

Experimental design consisted of conspecific and heterospecific matings between the species *D. mojavensis* and *D. arizonae* considering three biological replicates composed of 20 pooled dissected heads (Fig. 1). After trimming and filtering of sequence reads, we obtained an average of 20 million mapped reads across the 30 RNA-seq libraries. Minimum count filtering was applied independently to all different subfeatures (e.g. exon, junction, intron) at the beginning of each analysis performed. We found evidence for gene expression changes when comparing mated with virgin samples (Fig. 2a). These changes were consistently detected at different hierarchies of gene expression such as at gene-wide (*DE*, using *FDR = 0.05*) (Fig. 3) as well as at exon and junction features (*AS*, using *FDR = 0.01*), including up to 16% of the *AS* genes exhibiting differential *IR* (using *FDR = 0.05*). Although the *DE* - *AS* overlap never exceeded 5% of genes, as expected from their distinct molecular regulatory mechanisms, both generally followed similar patterns in postmating experiments (Fig. 2a). However, the relative contribution of *DE* and *AS* showed large variation across the different conditions of the experiment. The overlap of genes responding to con- vs heterospecific matings was very low for all experiments, ranging from 14 to 28% (Fig. 2a), indicating that copulation between either *♀Dmoj* or *♀Dari* with a heterospecific male induces a very different transcriptional response in female heads.

The female’s species (*♀Dmoj* or *♀Dari*) seems to define the main patterns for expression responses when crossing *D. mojavensis* and *D. arizonae*. It defines the strength of the con- vs heterospecific response (Fig. 3) as well as when such changes are induced in the heads (45 min vs 6 h postmating periods). *♀Dmoj* crosses exhibited a larger response at 45 min (Fig. 3a) and higher number of genes when compared to *♀Dari*, which then tended to decrease over time. *♀Dari* matings generated a response to the heterospecific matings that was slower and tended to increase over time, but decreased for female heads of conspecific matings (Figs. 2a and 3b).

The direction of expression changes in mated females compared to female virgin samples showed an overrepresentation of up- vs downregulated genes (Fig. 2b). Thus, *♀Dmoj* matings exhibited up to four times more upregulated than downregulated genes (Fig. 2b), while *♀Dari* matings had over twice as many downregulated genes as upregulated. The number of genes and the distribution of the expression response in *DE* genes were substantially perturbed by the heterospecific matings (Fig. 3). The response of *♀Dmoj* was stronger for conspecific matings in terms of the number of genes involved (Fig. 2b, Fig. 3) when compared to that of heterospecific matings, while *♀Dari* involved more genes in the heterospecific matings than that of the conspecific matings (Fig. 2b, Fig. 3). Expression of most of the significant *DE* genes common to con- and heterospecific responses was highly correlated, suggesting that these genes follow similar directions regardless of the species identity of the male (Fig. 3). However, a few of these genes show interesting and opposing patterns between con- and heterospecific matings, which make them additionally interesting candidates to further investigate in the context of the reproductive isolation between the cactophilic *Drosophila* species (Supplementary Tables 1S and 2S).

### Transcriptional correlation between crosses and postmating periods

**Fig. 4:**
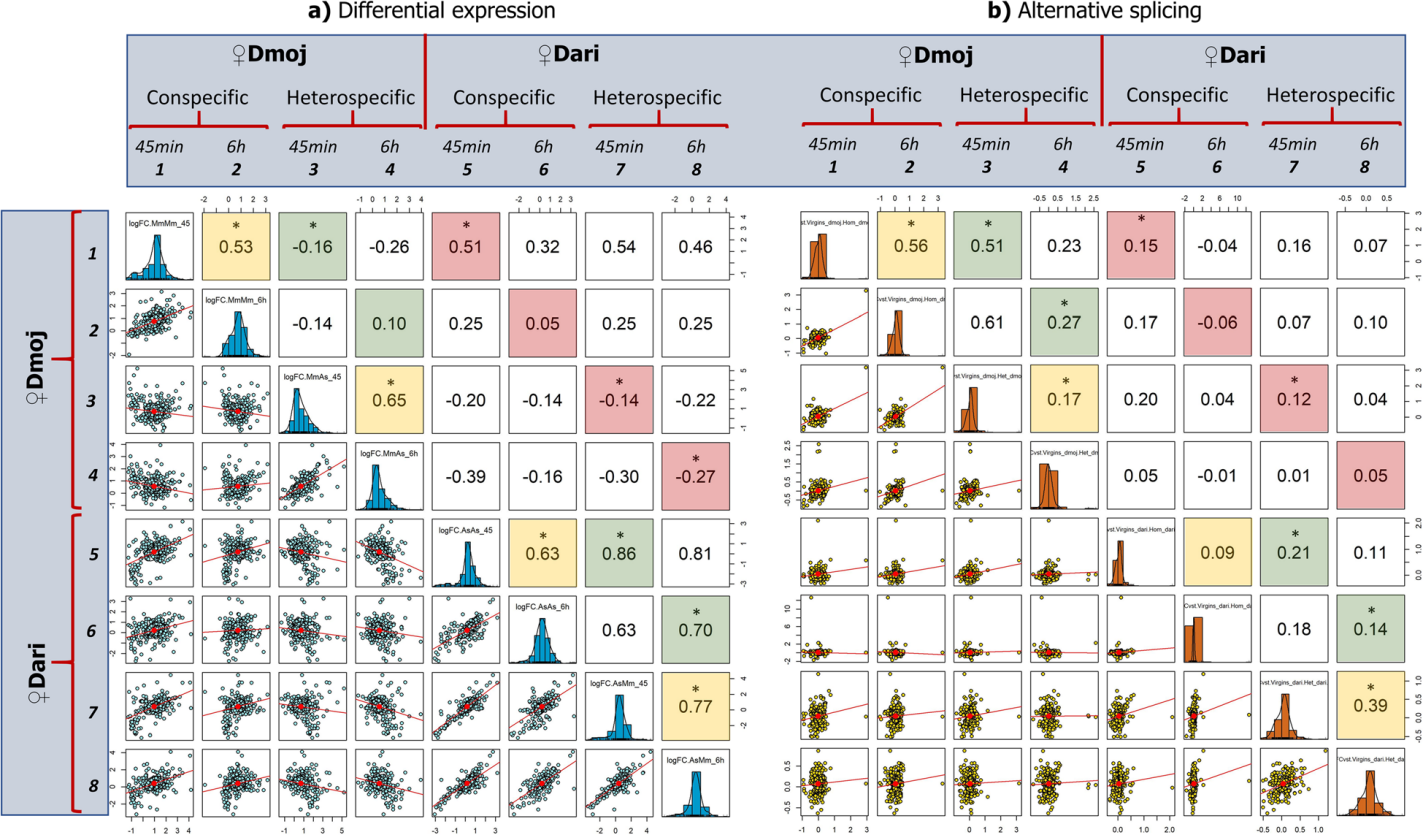
Transcriptional correlations between con- vs heterospecifically-mated females of *D. mojavensis* and *D. arizonae*. Pairwise correlation coefficient matrix (Spearman’s *ρ*) of relative gene fold expression (*log*_*2*_*FC*) was estimated only for genes with significant **a)** Differential expression (*FDR* < 0.05) and **b)** Alternative splicing (*FDR* < 0.01). Biologically meaningful correlations are highlighted for correlations: 45 min vs 6 h (yellow), con- vs heterospecific matings (green) and between species correlations (pink). Significant correlations (α < 0.05) are indicated with *

We next examined the level of transcriptional correlation between con- vs heterospecific matings for genes showing significant *DE* and *AS* responses. Overall, *DE* and *AS* correlations show similar tendencies, with a strong initial correlation between con- vs heterospecific matings at 45 min that tended to decrease substantially at 6 h (Fig. 4). One exception was the case of *♀Dmoj* (con vs hetero), where *DE* genes (Fig. 4a) showed very low correlation even at 45 min postmating (Spearman’s *ρ* = − 0.16), indicating substantial transcriptional perturbation in heterospecifically-mated females (Fig. 4a). *♀Dari* (con vs hetero) on the other hand showed a stronger transcriptional correlation at 45 min (Spearman’s *ρ* = 0.86) which then decreased at 6 h (Spearman’s *ρ* = 0.70). This pattern was opposite to that observed in *AS* genes (Fig. 4b), where *♀Dmoj* showed a strong con vs hetero correlation at 45 min, which was disrupted at 6 h, while transcriptional perturbation appeared earlier (at 45 min postmating) in *♀Dari* crosses (Fig. 4b).

We next investigated the level of correlation for genes responding to mating dynamics between the species (*♀Dmoj* vs *♀Dari*). With the exception of *DE* genes at 45 min (Fig. 4a), which showed a moderate correlation in the conspecific-mating response between the species (*♀Dmoj* con vs *♀Dari* con, Spearman’s *ρ* = 0.51), the rest of the comparisons (*DE* and *AS*) were not correlated between the species (con- or heterospecific) (Fig. 4a and b).

**Fig. 5:**
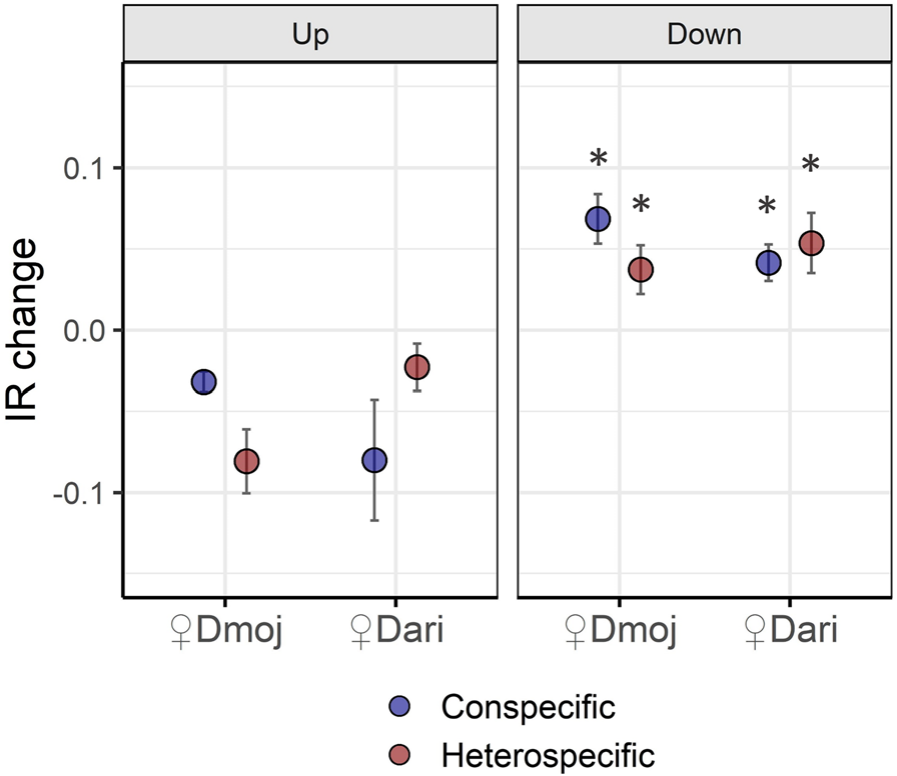
Intron retention change (*IR change*) as estimated for up- and down-regulated genes following con- and heterospecific matings between *D. mojavensis* and *D. arizonae*. *IR* change was estimated as the Euclidian distance between the *IR* rates of mated and virgin samples. All mating experiments showed significant increase of *IR* rate for down-regulated genes with respect to that of the up-regulated ones. All significant comparisons (α < 0.05) following *GLM* analysis are indicated with * in the “down” plot

Most of *AS* dynamics detected through *junctionSeq* reflected differential isoform regulation. However, the specific case of intron retention, as detected using *IRFinder,* more likely indicates gene regulation by nonsense-mediated decay (*NMD*) or a similar pathway. We tested this hypothesis as a possible mechanism of transcriptional response to mating by estimating intron retention changes between mated vs virgin samples (*IR change*), for up and down-regulated genes (Fig. 5). We discovered that *IR change* consistently increased for down-regulated genes, while it decreased for up-regulated genes in response to all mating experiments (Fig. 5). This finding is consistent with *IR* serving as a mechanism of gene expression downregulation.

### Evidence of positive selection in postmating responsive genes

**Fig. 6:**
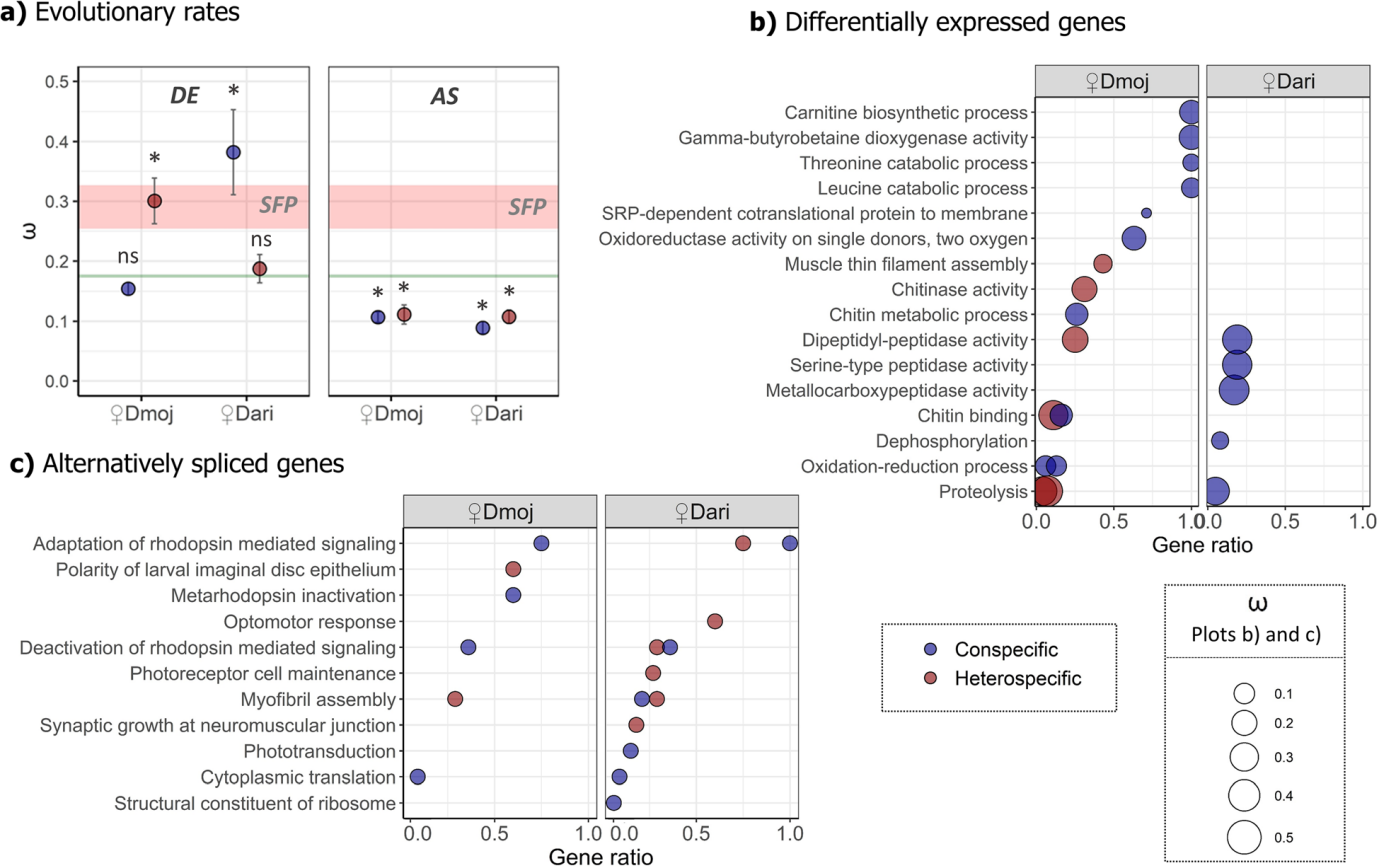
Evolutionary rates and functional analysis of significant *DE* and *AS* genes detected in head transcriptome of mated females between *D. mojavensis* and *D. arizonae*. **a** Average pairwise ω of *DE* and *AS* genes. The variation of ω for the genome background (green) and seminal fluid protein genes are indicated (*SFP* in pink). These genes are a subset of accessory gland-biased genes that contain a predicted signal sequence following Kelleher et al. [38]. Error bars represent standard error of the mean. Significant comparisons from genome background rates following *GLM* analysis (α < 0.05) are indicated with *. Functional analyses are shown for **b** *DE* and **c** *AS* genes, indicating gene ontology enrichment categories for con- and heterospecific matings between the species. The gene ratio of significantly detected genes within each enriched category is indicated (Gene ratio = significant genes in category / total number of genes in category). All significant comparisons with following *GLM* analysis are indicated with *

We investigated rates of molecular evolution (ω = *dN/dS*) of *DE* and *AS* genes for each experimental cross. Results on evolutionary rates revealed two main patterns of molecular evolution in these genes (Fig. 6a). Firstly, *AS* genes seem to evolve at a much lower evolutionary rate than *DE* genes, even lower than the genome background. Secondly, *DE* genes exhibited substantial differences in the evolutionary rates between both species and crosses. The average ω ratio was substantially higher than the genome background for conspecific matings in *D. arizonae* (ω = 0.38, Fig. 6a)*,* while *D. mojavensis* genes evolve at background rates. The heterospecific matings show exactly the opposite pattern, with *DE* genes from heterospecifically-mated *D. mojavensis* females evolving more rapidly than background (ω = 0.30), while heterospecifically-mated *D. arizonae DE* genes evolve at genome background rates (ω = 0.18, Fig. 6a). Rapidly evolving *DE* genes appear to change at similar rates or even higher than those of seminal fluid proteins (SFP) previously reported by Kelleher et al. [38] in *D. mojavensis* (Fig. 6).

### Functional specialization played by DE and AS genes

To analyze the functional pathways associated with female genes responding to mating experiments in female heads, we performed gene ontology (*GO*) enrichment analysis. We found that detected genes were enriched in four main functional pathways (Fig. 6b and c): i) nutrient homeostasis, ii) chitin metabolism, iii) photoreception and iv) muscle assembly (Fig. 6). Pathways associated with i) nutrient homeostasis are particularly interesting for their implications in the female postmating response. These pathways are all associated with amino acid balance in brains: L-Carnitine from Lysine and Methionine (*Carnitine biosynthetic process* and *Gamma-butyrobetaine dioxygenase activity*, Fig. 6b), and the production of Threonine and Leucine (*Threonine* and *Leucine biosynthetic process*, Fig. 6b). Furthermore, some of these metabolic functions have been previously detected in conspecific mating experiments in *D. melanogaster* [28, 39]. Physiological functions associated with these specific amino acids include energy balance in brain tissues (for Carnitine) [40, 41], insulin secretion following food intake, increasing cellular uptake of nutrients (for Leucine) [42] and female behaviors such as sleep suppression (for Threonine) [43, 44].

We found functional specialization between *DE* and *AS* genes, with *DE* (Fig. 6b) patterns being more associated with pathways of i) nutrient homeostasis and ii) chitin metabolism, while *AS* genes (Fig. 6c) were dominated by functional networks related to iii) photoreception and iv) muscle assembly. Both mechanisms of gene regulation showed dramatic functional differentiation between con- vs heterospecific crosses (Fig. 6b and c). Most of the enriched pathways were detected in a conspecific context, but only a subset remained significant in the heterospecific matings (Fig. 6b and c). Genes associated with i) nutrient homeostasis and iii) photoreception, activated in conspecific matings, were not activated in heterospecifically-mated *♀Dmoj*. Similarly, all proteolytic pathways activated in conspecific *♀Dari* were not activated in heterospecific matings. Genes that have been previously reported as related with the i) female behavior were exclusive to *D. mojavensis* and were not enriched in *D. arizonae* (Fig. 6b).

## Discussion

The cactophilic *D. mojavensis* and *D. arizonae* are promiscuous flies, mating multiple times a day in a laboratory setting, even to heterospecifics (*Diaz* et al *unpublished data*). Yet these species have remained isolated for ~ 0.5 My, suggesting the presence of multiple reproductive barriers preventing introgressive hybridization [36, 45, 46]. *PMPZ* isolation has been confirmed for crosses involving *D. mojavensis* females [21], where the reaction mass is more evident and molecular interactions in the female lower reproductive tract were found altered by the heterospecific ejaculate [8]. Here, we demonstrate that copulation induces substantial transcriptional changes in head tissues of females that are substantially perturbed when mating with a heterospecific male in both species. These changes compromise functional pathways important for the female postcopulatory physiology and behavior that normally would be expressed in conspecifically-mated females. Moreover, some of these genes evolve rapidly, which might have implications for the extent of sexual selection and sexual conflict [47].

Our results indicate that mating induces not only gene expression changes in female heads soon after mating, but also that a great part of the female postmating response involves a change in alternative splicing. The number of genes responding through *AS* often exceeded that of *DE* genes, but both mechanisms appear to be involved in the female postcopulatory response. We examined *AS* patterns caused by multiple mechanisms, including intron retention (*IR*) [18], when comparing mated vs virgin females. Differential usage of examined gene features showed substantial consequences for the postmating response that were not reflected in gene expression of head transcriptomes. The role of *AS* in the postmating response has not been previously evaluated, but is consistent with the complexity of interactions related to sexual traits [34]. Interestingly, in this study, genes experiencing *DE* or *AS* appear to be almost mutually exclusive (less than 5% overlap). Consequently, the female postmating response seems to target different functions through each of these mechanisms. *DE* genes are mainly linked to pathways of proteolysis and nutrient homeostasis, while *AS* genes are more related to those involved in photoreception and muscle assembly changes.

*IR* is a particular case of *AS* that, although it has been associated with some active functional changes, is most likely linked to negative gene regulation resulting from the degradation of mRNA by the nonsense-mediated mRNA decay (*NMD*) pathway [20, 48]. We demonstrate that the extent of *IR* is not only different between con- and heterospecific matings but also seems to be an active mechanism of gene regulation, as *IR* rates increased for down regulated genes, but decreased for up-regulated genes.

The transcriptional response to mating has been studied in a few insect species [9–16], showing biologically meaningful pathways common to the postmating response across different species. In fact, some of the mating-activated genes that we found in female heads are associated with functional pathways previously reported in different tissues and species. Proteolytic pathways for example, are within the most common and strongly activated genes that are part of the female response, found in both reproductive tissues and whole female bodies [10, 39, 49]. However, this is a complex reproductive response, given that a great part of the male ejaculate is also composed of a diverse cocktail of both proteases and their inhibitors [2].

A great array of proteases with diverse functions are associated with the postcopulatory female response [4, 12, 50]. Most of these are related to earlier postmating processes and molecular interactions occurring in the female tract [e.g. sperm storage and cleavage of seminal fluid proteins (*SFP*)] [50–52], but it is unclear whether the proteases or inhibitors expressed by the female are involved in the same functions. However, the lasting effects, even several days after mating, and the fact that they have been detected in several species, from insects to mammals [4, 52], suggest that these proteolytic cascades are involved in multiple functions of the whole organism mating response. In this study, the expression of proteolytic cascades we observed in heads of mated females does not seem connected to functions occurring in the female reproductive tract. One possibility is that some of these cascades are involved in protein degradation for amino acid related pathways and nutrient homeostasis, a hypothesis supported by our functional analysis. We identified two functional categories associated with Carnitine biosynthesis. This particular amino acid is known to start with the degradation of proteins containing N-methylated lysine by proteolytic cascades [53], with a major role in energy homeostasis in the nervous system [40, 41].

We detected several biosynthetic pathways associated with the production of specific amino acids important for nutrient homeostasis such as carnitine, threonine, and leucine. Although such pathways are not directly classified as behavioral or reproductive, some of their metabolic functions have been previously detected in conspecific experiments in *D. melanogaster* [28, 39]. These networks regulate energy balance [40, 41] and nutritional uptake in brain tissues [42], and have been directly linked to several postcopulatory behaviors, including circadian rhythms [43, 44], nutrient sensing, exploratory behavior for specific nutrient source, consumption and posterior oviposition [26, 27, 54, 55]. Consequently, mated *D. melanogaster* females experience a major switch in their diet following copulation [56], consuming more amino acids during the dark phase [26]. The mechanism of this behavioral switch has been linked to neuronal signaling triggered by *SP*-*SPR* dynamics in *D. melanogaster* [57–59].

Transcriptional dynamics detected in female heads of both species were substantially perturbed by heterospecific matings. Thus, changes of regulatory networks related to nutrient homeostasis in brain tissues were characteristic of the conspecific postmating response in *D. mojavensis*, while changes detected in *D. arizonae* females were dominated by proteolytic pathways. The biological function of genes, and the magnitude of their expression were perturbed following copulation with a heterospecific male. Heterospecifically-mated *D. mojavensis* did not activate nutrient-homeostasis- (*DE* genes)  or photoreception-related (*AS* genes) pathways, and activated proteolytic and some muscle assembly genes instead. In contrast, heterospecifically-mated *D. arizonae* females showed no enriched pathways for *DE* genes, suggesting strong functional perturbation. These results suggest that, soon after mating, expressed genes from the female reproductive tract likely modulate the expression of behavioral genes in distant tissues such as the female head [28, 39]. The mode and mechanism of signaling for these changes in insects is still an ongoing investigation. Given the distance and heterogeneity of the tissues involved, changes induced in the female head are assumed to be the product of altered interaction networks, influencing the physiology of other tissues [29, 60]. This is more an indirect effect of neurons associated with internal reproductive tissues, which may produce signaling molecules that influence gene expression at distant sites in the fly body [61]. However, evidence from *D. melanogaster* suggests that seminal fluid components like the *SP* rapidly circulate in the female’s hemolymph and has been found associated with brain tissues [32]. Furthermore, immediate post-copulatory transcriptional responses in the nervous system could also be due to social interactions that occur during courtship, as has been shown in *D. melanogaster* with males that court but fail to copulate [62]. Further research is needed to disentangle the underlying mechanisms causing the activation of genes outside of the female reproductive tract and their involvement in the post-copulatory response.

Although female reproductive tract genes involved in the postmating response have only been investigated in a few species, results from *D. mojavensis* and *D. virilis* [45, 49, 63] indicate that these genes do not always evolve as rapidly as male reproductive genes. Here, we found that *DE* genes in female heads are evolving rapidly in *D. arizonae*, but not in *D. mojavensis*. Conspecific genes detected in *D. arizonae* are related to different proteolytic pathways and showed evolutionary rates even higher than *SFP* genes previously detected in these species [38]. These pathways were not enriched in *D. mojavensis* females, but some were activated when mated with *D. arizonae* males. Substantial postmating transcriptional differences in rapidly evolving genes connected to female behavior might have implications for the reproductive barriers between *D. mojavensis* and *D. arizonae*. For example, one possibility that could influence the expression pattern detected in female heads is the difference between the species in signals elicited during courtship [64]. In contrast to *D. melanogaster,* where courtship is longer and involves visual displays in front of the female [65, 66], courtship in cactophilic *Drosophila* likely involves chemical and auditory cues, as it occurs largely behind the female [64]. In addition, we have recently observed that *D. mojavensis* females are more likely to remate than *D. arizonae* females (*Diaz* et al. *unpublished data*). Differences in these courtship signals between *D. mojavensis* and *D. arizonae* may elicit altered transcriptional responses in female heads.

As opposed to *DE* genes*, AS* genes exhibited evolutionary rates even lower than the genome background. These genes were linked to very specific functions of highly conserved genes. Therefore, this could be due to the conservation of those specific gene functions in these species, but more likely reflects a general trend in the molecular evolution of *AS* genes [67]. To date, the role of *AS* in the female postmating response has not previously evaluated in insect species, nor the relationship between *AS* and molecular evolution in *Drosophila*. However, this same result was also previously found when investigating *AS* genes across the mouse and human genomes [67], suggesting that the level of alternative splicing and molecular evolution at the sequence level are negatively correlated. Constitutive exons of alternative isoforms tend to evolve faster than newly alternatively spliced exons [67, 68]. It is unclear how these heterogenic patterns would affect gene-wide molecular evolution or specific gene families in insects, but highly constrained exons could decrease rates of molecular evolution at the gene level. Alternatively, spliced genes may also be more pleiotropic, as alternative isoforms can evolve without major changes in sequence through functional specialization of different exon arrays.

## Conclusions

The female postmating response is a complex process that involves the general female physiology as well as strong tissue-specific transcriptional changes with variation in strength and direction [39]. By implementing an integrated transcriptional analysis in head tissues of mated females between *D. mojavensis* and *D. arizonae*, we were able to reveal previously unknown roles played by *DE*, *AS* and *IR*, and how these dynamics are integrated through functional specializations within the female postmating response. Substantial transcriptional perturbations resulting from heterospecific matings suggest a previously unknown role of these genes in postmating barriers that rely on female behavioral changes triggered during copulation. The consequences of mating interactions between the sexes have generally considered genes that are more directly linked to reproduction (i.e. *SFP* and female tract genes) [47]. We provide evidence showing that differentially expressed genes from distant head tissues evolve at rates that are similar, or even higher, than those of male reproductive genes. Given the functions of genes involved, these changes might be costly to the female, affecting not only fertilization, but also could alter hybrid performance (if fertilization succeeds) as well as subsequent nutritional and egg-laying decisions by the female. These types of changes have been detected in a few species [69–71], but their transcriptional basis and evolutionary rates have remained unknown. Our results indicate that transcriptional perturbation following heterospecific mating extends beyond the female reproductive tract. The extent of these interactions might be larger than previously thought, opening the door to investigate the link between head transcriptomes and reproductive barriers between species.

## Methods

### Samples and mating experiments

All experiments were carried out using *D. mojavensis* and *D. arizonae* isofemale lines originally collected from Anza Borrego Desert State Park, Borrego Springs, CA (in 2002) and Guaymas, Sonora, Mexico (in 2000), respectively. Inbred lines were held at 25 °C, under 12:12 h light:dark cycle and controlled density conditions in 8-dram glass vials with banana-molasses media [72] for all stocks and experiments. Experimental design consisted of conspecific (*♀ D. mojavensis* x *♂ D. mojavensis* and *♀ D. arizonae* x *♂ D. arizonae*) and heterospecific matings between the species (*♀ D. mojavensis* x *♂ D. arizonae* and *♀ D. arizonae* x *♂ D. mojavensis*) (Fig. 1). We refer to the reciprocal mating as *♀Dmoj* for matings involving *D. mojavensis* females and *♀Dari* for those involving *D. arizonae* females. All mating experiments were performed using 7–10-day old virgin flies. Males and females were paired in vials and were constantly inspected for copulation events during a 2-h window immediately after the incubator lights turned on. Males were removed from vials after copulation and females were kept in the vial until the specified postmating period was reached (45 min or 6 h), when heads were collected from both mated and control virgin females (Fig. 1). Groups of 20 dissected heads were pooled for each sample and three biological replicates were collected per experimental cross, which generated 15 samples for each direction of the cross (*♀Dmoj* and *♀Dari* -con/−het) for a total of 30 samples. All tissues were placed immediately in TRIzol and kept at − 80 °C until total RNA extractions.

### RNA extraction, cDNA library construction and sequencing

Total RNA was extracted using Direct-zol RNA kit (Zymo Research). Both RNA quality and quantity were inspected on a Bioanalyzer (Applied Biosystems/Ambion). cDNA libraries were created using KAPA Stranded mRNA-Seq Kit according to the manufacturer’s instructions. The 30 RNA-seq libraries were sequenced at Novogene Inc. using the HiSeq SBS v4 High Output Kit on Illumina platform flow cells with runs of 2 × 150 bp paired-end reads. Illumina’s HiSeq Control Software and CASAVA software (Illumina, Inc.) were used for base calling and sample demultiplexing.

### Sequence trimming and mapping

Nearly 700 million total paired-end read sequences were obtained from the Illumina runs, ranging from 16 to 27 million reads per sample. Reads were trimmed for quality and adapter sequences were removed using a minimum quality base of Q = 20 and minimum read length of 50 bp using the software Trimmomatic [73]. Trimmed reads were then mapped to corresponding reference genomes using splice-aware mapper *GSNAP* [74] with the option of new splice events detection. The *D. mojavensis* reference genome was used for samples involving *♀Dmoj*, while *D. arizonae* was used as reference for *♀Dari* samples. Generated *sam* files were converted to *bam* format after indexing and filtering for a minimum mapping quality of MQ = 20 using SAMtools [75]. These mapping results were then used for all differential expression and alternative splicing downstream pipelines.

### Reference genomes

Template based genomes were used for mapping RNAseq reads. For *D. mojavensis*, the assembly from [76] (SRP190536) was used with updated annotations retrieved from FlyBase version FB2016_05 [77]. A template genome version of *D. arizonae* (https://osf.io/ukexv/?view_only=7e20375d605040e4add37f707cbabaf4) was assembled using the same method as *D. mojavensis* in [76] with paired-end and mate pair Illumina reads from [78] (SRP278895).

### Differential expression - DE

We created a gene level read count matrix for all samples using *featureCounts* [79]. The read count matrix was filtered for a minimum count cutoff = 3 cpm over at least two replicates per comparable group. All *DE* analyses were performed using the R package *edgeR* [80] after *TMM* library normalization. Normalized counts were analyzed by Generalized Linear Models (*GLM*) assuming a negative binomial model of read counts, followed by *DE* analyses. All comparisons were performed between mated females (con- and heterospecific mating) and virgin females (*♀ Dmoj* and *♀ Dari*) at postmating periods (45 min and 6 h) using three biological replicates per condition. Features with a false-discovery rate (*FDR*) corrected *p*-value < 0.05 [81] and a log_2_-fold-change threshold of > 1.0 were considered significant.

### Alternative splicing - AS

We used the *JunctionSeq* [82] pipeline in order to detect genome-wide patterns of alternative spliced genes. *AS* is defined as the relative regulation of isoforms belonging to a multi-isoform gene with respect to a given biological condition [83]. The pipeline is based on differential usage calculated from both exon and junction feature coverages. The pipeline relies on the originally implemented method in *DEXSeq* [84], which tested differential usage of annotated exons, but extended to splice junctions usage and both annotated and non-annotated splicing events. A new flattened *GTF* annotation file where overlapping features are not allowed was first generated using *QoRTs* [83]. All overlapping genes were merged as composed by a flat set of non-overlapping exons and splice junctions with unique identifiers. *QoRTs* was also used to generate a read count matrix for *AS* analysis, including three types of read counts per gene as estimated by exons, junction and gene level counts. The generated count matrix was then used by *JunctionSeq* R package [82] to estimate differential exon and junction usage with respect to gene-wide expression. No read was counted more than once in the model since exon and junction dispersions are then fitted independently. As for differential expression, alternatively spliced genes were detected if at least one exon or splice junction was differentially used between mated females (con- and heterospecific mating) and virgin females (*♀Dmoj* and *♀Dari*) at postmating periods (45 min and 6 h) using three biological replicates. Only features with *p*-values < 0.01 after FDR correction were considered significant.

### Intron retention rates - IR

Intron retention is a specific type of *AS* that is not necessarily captured by *JunctionSeq* and can have different biological implications that help to better explain expression changes. An intron can be retained in the final mature *mRNA*, coding for a new function [85, 86] or a nonfunctional transcript that is degraded by nonsense-mediated decay (*NMD*) [87]. We investigated whether postmating *AS* events also involve mechanisms of intron retention using the *IRFinder* pipeline [88]. A new reference annotation was built by removing all overlapping features present in the same strain sense of individual introns and then unique identifiers were assigned to each flattened exon. Only regions with high mapping scores as estimated through simulated reads across the genome were identified and included in the flattened annotation file. A read count matrix with all reads overlapping splice junctions was generated and *IR* rates were estimated as: *junction reads* / (*junction reads* + *intronic reads*) for each sample. The count matrix was then used by *IRFinder* R package [88] in order to estimate *GLM*. This method is used to test the fold change of *IR* between biological conditions using the *DESeq2* R package framework [89]. Genes with differential *IR* were then detected if least one intron was differentially retained between mated females (con- and heterospecific mating) and virgin females (*♀Dmoj* and *♀Dari*) at postmating periods (45 min and 6 h) using three biological replicates. Only features with p-values < 0.01 after FDR correction were considered significant.

### Statistical analysis of genes under DE, AS and IR

A Spearman’s correlation matrix comparing relative expression levels of significant *DE* genes was generated in order to investigate the relationship of gene expression changes between con- vs heterospecific matings as well as between postmating periods (45 min vs 6 h) and the two directions of the cross (*♀Dmoj* vs *♀Dari*). Because *IR* changes are more likely linked to mechanisms of downregulation by transcript degradation, we tested this hypothesis by estimating *IR* changes between mated vs virgin samples–*IR change*, while comparing up vs down-regulated genes. A *GLM* analysis was performed using categories of up and down regulation as independent variables and the level of *IR* change as the dependent variable for each mating experiment. *GLM* analysis was performed after square root transformation to normalize the error distribution and to achieve homoscedasticity.

### Functional and evolutionary analyses

Overrepresentation of specific categories of biological functions were then investigated for *DE* and *AS* genes using *GOseq* R package framework [90]. Additionally, we investigated signatures of positive selection on genes responding to con- vs heterospecific matings as well as for each of the overrepresented categories detected. For this, we estimated evolutionary rates (w = *d*_*n*_*/d*_*s*_) using *codeml*, part of *PAML* 4.9 [91]. *CDS* alignments between *D. mojavensis* and *D. arizonae* were produced with *MUSCLE* 3.8.31 [92]. Any alignments with internal stop codons or frameshifts were removed before analysis. *Codeml* was run using model *0* with default values. Raw synonymous and nonsynonymous polymorphism counts were generated with *KaKs* Calculator 1.2 [93]. We further extracted putative seminal fluid genes identified in a proteomic analysis of *D. mojavensis* male accessory glands [38] and compared their rate of molecular evolution with those that were differentially regulated in heads. A *GLM* analysis was performed to compare the average evolutionary rates under each mating experiment against the genome background rates for each mating experiment. *GLM* analysis was performed after square root transformation to normalize the error distribution and to achieve homoscedasticity.

## Supplementary Information

**Additional file 1: Table S1.** Differentially expressed genes in head transcriptomes (FDR with alpha = 0.05) of females following con- and heterospecific matings between *D. mojavensis and D. arizonae*. Results are showing significant DE genes for each female species (Dmoj and Dari) and postmating period (45 min and 6 h). Significant (Con- or Heterospecific) and overlapping genes (Consp. and Hetero.) are indicated. Overlapping genes showing substantial differences between con- and heterospecific crosses are indicated (Consp. and Hetero. Diferent responses). Gene IDs and Gene symbols are based on *D. mojavensis* genome as extracted from *D. melanogaster* orthologous. Gene IDs based on *D. mojavensis* genome, while Gene symbols are extracted from *D. melanogaster* orthologous. (NA: not applicable). **Table S2.** Alternatively spliced genes in head transcriptomes (FDR with alpha = 0.01) of females following con- and heterospecific matings between *D. mojavensis* and *D. arizonae.* Results are showing significant AS genes for each female species (Dmoj and Dari) and postmating period (45 min and 6 h). Significant (Con- or Heterospecific) and overlapping genes (Consp. and Hetero.) are indicated. Gene IDs based on *D. mojavensis* genome, while Gene symbols are extracted from *D. melanogaster* orthologous. (NA: not applicable).

## Data Availability

A template genome version of *D. arizonae* and *D. mojavensis* was deposited in public repository (https://osf.io/ukexv/?view_only=7e20375d605040e4add37f707cbabaf4), while all RNAs-seq reads have been deposited in the Sequence Read Archive under accession number (https://dataview.ncbi.nlm.nih.gov/object/PRJNA693826?reviewer=krjn5pt4ejuuehnlhbhhkeiccs).

## Abbreviations

*DE*: Differential expression
*AS*: Alternative splicing
*IR*: Intron retention
*♀Dmoj*: female *D. mojavensis*
*♀Dari*: female *D. arizonae*

## References

[1] Dimijian GG (2005). Evolution of sexuality: biology and behavior. Baylor Univ Med Cent Proc.

[2] Wolfner MF (2009). Battle and ballet: molecular interactions between the sexes in drosophila. J Hered.

[3] Manier MK, Lüpold S, Belote JM, Starmer WT, Berben KS, Ala-Honkola O, Collins WF, Pitnick S (2013). Postcopulatory sexual selection generates speciation phenotypes in drosophila. Curr Biol.

[4] Avila FW, Sirot LK, LaFlamme BA, Rubinstein CD, Wolfner MF (2011). Insect seminal fluid proteins: identification and function. Annu Rev Entomol.

[5] Ahmed-Braimah YH, Unckless RL, Clark AG (2017). Evolutionary dynamics of male reproductive genes in the drosophila virilis subgroup. G3 genes, genomes. Genet..

[6] Mueller JL, Ravi Ram K, McGraw LA, Bloch Qazi MC, Siggia ED, Clark AG (2005). Cross-species comparison of Drosophila male accessory gland protein genes. Genetics..

[7] Dottorini T, Nicolaides L, Ranson H, Rogers DW, Crisanti A, Catteruccia F (2007). A genome-wide analysis in Anopheles gambiae mosquitoes reveals 46 male accessory gland genes, possible modulators of female behavior. Proc Natl Acad Sci U S A.

[8] Bono JM, Matzkin LM, Kelleher ES, Markow TA (2011). Postmating transcriptional changes in reproductive tracts of con- and heterospecifically mated Drosophila mojavensis females. Proc Natl Acad Sci U S A.

[9] Liu PC, Hao DJ. Behavioural and transcriptional changes in post-mating females of an egg parasitoid wasp species. R Soc Open Sci. 2019;6(1). 10.1098/rsos.181453. 10.1098/rsos.181453 PMC6366167 30800387

[10] Mack PD, Kapelnikov A, Heifetz Y, Bender M (2006). Mating-responsive genes in reproductive tissues of female Drosophila melanogaster. Proc Natl Acad Sci U S A.

[11] Thailayil J, Gabrieli P, Caputo B, Bascuñán P, South A, Diabate A (2018). Analysis of natural female post-mating responses of Anopheles gambiae and Anopheles coluzzii unravels similarities and differences in their reproductive ecology. Sci Rep.

[12] Alfonso-Parra C, Ahmed-Braimah YH, Degner EC, Avila FW, Villarreal SM, Pleiss JA (2016). Mating-induced Transcriptome changes in the reproductive tract of female Aedes aegypti. PLoS Negl Trop Dis.

[13] Kocher SD, Richard FJ, Tarpy DR, Grozinger CM (2008). Genomic analysis of post-mating changes in the honey bee queen (Apis mellifera). BMC Genomics.

[14] Gao B, Song XQ, Yu H, Fu DY, Xu J, Ye H. Mating-induced differential expression in genes related to reproduction and immunity in Spodoptera litura (Lepidoptera: Noctuidae) female moths. J Insect Sci. 2020;20(1). 10.1093/jisesa/ieaa003. 10.1093/jisesa/ieaa003 PMC7039226 32092133

[15] Fowler EK, Bradley T, Moxon S, Chapman T (2019). Divergence in transcriptional and regulatory responses to mating in male and female Fruitflies. Sci Rep.

[16] Al-Wathiqui N, Dopman EB, Lewis SM (2016). Postmating transcriptional changes in the female reproductive tract of the European corn borer moth. Insect Mol Biol.

[17] Telonis-Scott M, Kopp A, Wayne ML, Nuzhdin SV, McIntyre LM (2009). Sex-specific splicing in Drosophila: widespread occurrence, tissue specificity and evolutionary conservation. Genetics..

[18] Venables JP, Tazi J, Juge F (2012). Regulated functional alternative splicing in Drosophila. Nucleic Acids Res.

[19] Gibilisco L, Zhou Q, Mahajan S, Bachtrog D (2016). Alternative splicing within and between Drosophila species, sexes, tissues, and developmental stages. PLoS Genet.

[20] Wang M, Branco AT, Lemos B (2018). The Y chromosome modulates splicing and sex-biased intron retention rates in Drosophila. Genetics..

[21] Kelleher ES, Markow TA (2007). Reproductive tract interactions contribute to isolation in Drosophila. Fly (Austin).

[22] Bloch Qazi MC, Wolfner MF (2003). An early role for the Drosophila melanogaster male seminal protein Acp36DE in female sperm storage. J Exp Biol.

[23] Neubaum DM, Wolfner MF (1999). Mated Drosophila melanogaster females require a seminal fluid protein, Acp36DE, to store sperm efficiently. Genetics..

[24] Sirot LK, Wolfner MF, Wigby S (2011). Protein-specific manipulation of ejaculate composition in response to female mating status in Drosophila melanogaster. Proc Natl Acad Sci U S A.

[25] Hollis B, Koppik M, Wensing KU, Ruhmann H, Genzoni E, Erkosar B, Kawecki TJ, Fricke C, Keller L (2019). Sexual conflict drives male manipulation of female postmating responses in Drosophila melanogaster. Proc Natl Acad Sci U S A.

[26] Uchizono S, Tabuki Y, Kawaguchi N, Tanimura T, Itoh TQ (2017). Mated Drosophila melanogaster females consume more amino acids during the dark phase. PLoS One.

[27] Corrales-Carvajal VM, Faisal AA, Ribeiro C (2016). Internal states drive nutrient homeostasis by modulating exploration-exploitation trade-off. Elife..

[28] Dalton JE, Kacheria TS, Knott SRV, Lebo MS, Nishitani A, Sanders LE, et al. Dynamic, mating-induced gene expression changes in female head and brain tissues of Drosophila melanogaster. BMC Genomics. 2010;11(1). 10.1186/1471-2164-11-541. 10.1186/1471-2164-11-541 PMC3091690 20925960

[29] Swanson WJ (2003). Sex peptide and the sperm effect in Drosophila melanogaster. Proc Natl Acad Sci U S A.

[30] Peng J, Chen S, Bu S, Liu H, Honegger T, Kubli E (2005). Gradual release of sperm bound sex-peptide controls female Postmating behavior in Drosophila. Curr Biol.

[31] Ram KR, Wolfner MF (2007). Sustained post-mating response in Drosophila melanogaster requires multiple seminal fluid proteins. PLoS Genet.

[32] Yapici N, Kim YJ, Ribeiro C, Dickson BJ (2008). A receptor that mediates the post-mating switch in Drosophila reproductive behaviour. Nature..

[33] Delbare SYN, Chow CY, Wolfner MF, Clark AG, Wilson SM (2017). Roles of female and male genotype in post-mating responses in Drosophila melanogaster. J Hered..

[34] Swanson WJ, Vacquier VD (2002). The rapid evolution of reproductive proteins. Genetics..

[35] Matzkin LM (2014). Ecological genomics of host shifts in *Drosophila mojavensis*. Adv Exp Med Biol.

[36] Knowles LL, Markow TA (2001). Sexually antagonistic coevolution of a postmating-prezygotic reproductive character in desert Drosophila. Proc Natl Acad Sci U S A.

[37] Lopez-Maestre H, Carnelossi EAG, Lacroix V, Burlet N, Mugat B, Chambeyron S, Carareto CMA, Vieira C Identification of misexpressed genetic elements in hybrids between Drosophila-related species. Sci Rep 2017;7 December 2016:1–13. doi:10.1038/srep40618. 10.1038/srep40618 PMC5238404 28091568

[38] Kelleher ES, Watts TD, LaFlamme BA, Haynes PA, Markow TA (2009). Proteomic analysis of Drosophila mojavensis male accessory glands suggests novel classes of seminal fluid proteins. Insect Biochem Mol Biol.

[39] Newell NR, Ray S, Dalton JE, Fortier JC, Kao JY, Chang PL (2020). The drosophila post-mating response: gene expression and behavioral changes reveal perdurance and variation in cross-tissue interactions. G3 genes, genomes. Genet..

[40] Laranjeira A, Schulz J, Dotti CG (2016). Genes related to fatty acid β-oxidation play a role in the functional decline of the drosophila brain with age. PLoS One.

[41] Schulz JG, Laranjeira A, Van Huffel L, Gärtner A, Vilain S, Bastianen J (2015). Glial β-Oxidation regulates drosophila energy metabolism. Sci Rep.

[42] Ziegler AB, Manière G, Grosjean Y (2018). JhI-21 plays a role in Drosophila insulin-like peptide release from larval IPCs via leucine transport. Sci Rep.

[43] Sonn JY, Lee J, Sung MK, Ri H, Choi JK, Lim C, Choe J (2018). Serine metabolism in the brain regulates starvation-induced sleep suppression in Drosophila melanogaster. Proc Natl Acad Sci U S A.

[44] Ki Y, Lim C (2019). Sleep-promoting effects of threonine link amino acid metabolism in Drosophila neuron to GABAergic control of sleep drive. Elife..

[45] Kelleher ES, Swanson WJ, Markow TA (2007). Gene duplication and adaptive evolution of digestive proteases in Drosophila arizonae female reproductive tracts. PLoS Genet.

[46] Machado CA, Matzkin LM, Reed LK, Markow TA (2007). Multilocus nuclear sequences reveal intra- and interspecific relationships among chromosomally polymorphic species of cactophilic Drosophila. Mol Ecol.

[47] Dapper AL, Wade MJ (2020). Relaxed selection and the rapid evolution of reproductive genes. Trends Genet.

[48] Graveley BR, Brooks AN, Carlson JW, Duff MO, Landolin JM, Yang L, Artieri CG, van Baren MJ, Boley N, Booth BW, Brown JB, Cherbas L, Davis CA, Dobin A, Li R, Lin W, Malone JH, Mattiuzzo NR, Miller D, Sturgill D, Tuch BB, Zaleski C, Zhang D, Blanchette M, Dudoit S, Eads B, Green RE, Hammonds A, Jiang L, Kapranov P, Langton L, Perrimon N, Sandler JE, Wan KH, Willingham A, Zhang Y, Zou Y, Andrews J, Bickel PJ, Brenner SE, Brent MR, Cherbas P, Gingeras TR, Hoskins RA, Kaufman TC, Oliver B, Celniker SE (2011). The developmental transcriptome of Drosophila melanogaster. Nature..

[49] Ahmed-braimah YH, Wolfner MF, Clark AG. Differences in post-mating transcriptional responses between conspecific and heterospecific matings in *Drosophila*. Mol Biol Evol. 2021;38(3):986–99. 10.1093/molbev/msaa264 PMC7947788 33035303

[50] Karr TL (2007). Fruit flies and the sperm proteome. Hum Mol Genet.

[51] Sitnik J, Gligorov D, Maeda R, Karch F, Wolfner MF (2016). The female post-mating response requires genes expressed in the secondary cells of the male accessory gland in Drosophila melanogaster. Genetics..

[52] LaFlamme BA, Ravi Ram K, Wolfner MF (2012). The Drosophila melanogaster seminal fluid protease “Seminase” regulates proteolytic and post-mating reproductive processes. PLoS Genet.

[53] Strijbis K, Vaz FM, Distel B (2010). Enzymology of the carnitine biosynthesis pathway. IUBMB Life.

[54] Hang WG, Ming WL (2019). Recent advances in the neural regulation of feeding behavior in adult Drosophila. J Zhejiang Univ Sci B.

[55] Owusu-Ansah E, Perrimon N (2014). Modeling metabolic homeostasis and nutrient sensing in Drosophila: implications for aging and metabolic diseases. DMM Dis Model Mech.

[56] Kubli E (2010). Sexual behavior: dietary food switch induced by sex. Curr Biol.

[57] Ribeiro C, Dickson BJ (2010). Sex peptide receptor and neuronal TOR/S6K signaling modulate nutrient balancing in Drosophila. Curr Biol.

[58] Bowman E, Tatar M (2016). Reproduction regulates Drosophila nutrient intake through independent effects of egg production and sex peptide: implications for aging. Nutr Heal Aging.

[59] Gioti A, Wigby S, Wertheim B, Schuster E, Martinez P, Pennington CJ, Partridge L, Chapman T (2012). Sex peptide of Drosophila melanogaster males is a global regulator of reproductive processes in females. Proc R Soc B Biol Sci.

[60] Yang C, Rumpf S, Xiang Y, Gordon MD, Song W, Jan Y (2009). Control of the Postmating behavioral switch in Drosophila females by internal sensory neurons. Neuron..

[61] Häsemeyer M, Yapici N, Heberlein U, Dickson BJ (2009). Sensory neurons in the Drosophila genital tract regulate female reproductive behavior. Neuron..

[62] Carney GE (2007). A rapid genome-wide response to Drosophila melanogaster social interactions. BMC Genomics.

[63] Bono JM, Matzkin LM, Hoang K, Brandsmeier L (2015). Molecular evolution of candidate genes involved in post-mating-prezygotic reproductive isolation. J Evol Biol.

[64] Markow TA (1981). Courtship behavior and control of reproductive isolation between Drosophila mojavensis and Drosophila arizonensis. Evolution (N Y).

[65] Pavlou HJ, Goodwin SF. Courtship behavior in *Drosophila melanogaster*: Towards a “courtship connectome.” Curr Opin Neurobiol 2013;23:76–83. 10.1016/j.conb.2012.09.002, 1. 10.1016/j.conb.2012.09.002 PMC3563961 23021897

[66] Duhart JM, Baccini V, Zhang Y, Machado DR, Koh K. Modulation of sleep-courtship balance by nutritional status in <i>Drosophila<\i>. bioRxiv. 2020;9:1–23. 10.7554/eLife.60853 PMC7609064 33084567

[67] Cusack BP, Wolfe KH (2005). Changes in alternative splicing of human and mouse genes are accompanied by faster evolution of constitutive exons. Mol Biol Evol.

[68] Modrek B, Lee CJ (2003). Alternative splicing in the human, mouse and rat genomes is associated with an increased frequency of exon creation and/or loss. Nat Genet.

[69] Matute DR (2014). The magnitude of behavioral isolation is affected by characteristics of the mating community. Ecol Evol.

[70] McLain DK, Pratt AE (1999). The cost of sexual coercion and heterospecific sexual harassment on the fecundity of a host-specific, seed-eating insect (Neacoryphus bicrucis). Behav Ecol Sociobiol.

[71] Quinõnes-Lebrón SG, Kralj-Fišer S, Gregoric M, Lokovšek T, Candek K, Haddad CR (2016). Potential costs of heterospecific sexual interactions in golden orbweb spiders (Nephila spp.). Sci Rep.

[72] Coleman JM, Benowitz KM, Jost AG, Matzkin LM (2018). Behavioral evolution accompanying host shifts in cactophilic *Drosophila* larvae. Ecol Evol..

[73] Bolger AM, Lohse M, Usadel B (2014). Trimmomatic: a flexible trimmer for Illumina sequence data. Bioinformatics..

[74] Wu TD, Nacu S (2010). Fast and SNP-tolerant detection of complex variants and splicing in short reads. Bioinformatics..

[75] Li H, Handsaker B, Wysoker A, Fennell T, Ruan J, Homer N, Marth G, Abecasis G, Durbin R, 1000 Genome Project Data Processing Subgroup (2009). The sequence alignment/map format and SAMtools. Bioinformatics..

[76] Allan CW, Matzkin LM (2019). Genomic analysis of the four ecologically distinct cactus host populations of Drosophila mojavensis. BMC Genomics.

[77] Gramates LS, Marygold SJ, Dos Santos G, Urbano JM, Antonazzo G, Matthews BB (2017). FlyBase at 25: looking to the future. Nucleic Acids Res.

[78] Sanchez-Flores A, Peñaloza F, Carpinteyro-Ponce J, Nazario-Yepiz N, Abreu-Goodger C, Machado CA (2016). Genome evolution in three species of cactophilic drosophila. G3 genes, genomes. Genet..

[79] Liao Y, Smyth GK, Shi W (2014). featureCounts: an efficient general purpose program for assigning sequence reads to genomic features. Bioinformatics..

[80] Robinson MD, McCarthy DJ, Smyth GK (2009). edgeR: a bioconductor package for differential expression analysis of digital gene expression data. Bioinformatics..

[81] Benjamini Y, Hochberg Y (1995). Controlling the false discovery rate: a practical and powerful approach to multiple testing. J R Stat Soc.

[82] Hartley SW, Mullikin JC (2016). Detection and visualization of differential splicing in RNA-Seq data with JunctionSeq. Nucleic Acids Res.

[83] Hartley SW, Mullikin JC (2015). QoRTs: a comprehensive toolset for quality control and data processing of RNA-Seq experiments. BMC Bioinformatics.

[84] Anders S, Reyes A, Huber W (2012). Detecting differential usage of exons from RNA-seq data. Genome Res.

[85] Jacob AG, Smith CWJ (2017). Intron retention as a component of regulated gene expression programs. Hum Genet.

[86] Monteuuis G, Wong JJL, Bailey CG, Schmitz U, Rasko JEJ (2019). The changing paradigm of intron retention: regulation, ramifications and recipes. Nucleic Acids Res.

[87] Farlow A, Meduri E, Dolezal M, Hua L, Schlötterer C (2010). Nonsense-mediated decay enables intron gain in Drosophila. PLoS Genet.

[88] Middleton R, Gao D, Thomas A, Singh B, Au A, Wong JJL, Bomane A, Cosson B, Eyras E, Rasko JEJ, Ritchie W (2017). IRFinder: assessing the impact of intron retention on mammalian gene expression. Genome Biol.

[89] Love MI, Huber W, Anders S (2014). Moderated estimation of fold change and dispersion for RNA-seq data with DESeq2. Genome Biol.

[90] Young MD, Wakefield MJ, Smyth GK, Oshlack A (2010). Gene ontology analysis for RNA-seq: accounting for selection bias. Genome Biol.

[91] Yang Z (2007). PAML 4: phylogenetic analysis by maximum likelihood. Mol Biol Evol.

[92] Edgar RC (2004). MUSCLE: multiple sequence alignment with high accuracy and high throughput. Nucleic Acids Res.

[93] Zhang Z, Li J, Zhao X-Q, Wang J, Gane K-SW YJ (2006). KaKs_Calculator: calculating Ka and Ks through model selection and model averaging. Genomics Proteomics Bioinforma.

